# Supplementary material for: Multicenter Evaluation of Rapid BACpro® II for the Accurate Identification of Microorganisms Directly from Blood Cultures Using MALDI-TOF MS
Source: Diagnostics (Basel). 2021 Dec 1;11(12):2251. doi: 10.3390/diagnostics11122251 (PMC8700617; doi:10.3390/diagnostics11122251)
Supplement: Supplementary file 1 [file diagnostics-11-02251-s001.zip › diagnostics-1447244-supplementary.pdf]

	n	CHUAC						n	CHUV						n	HGM						n	OUH					
		S >2.0	(%)	1.7 < S < 1.99	(%)	S < 1.7	(%)		S >2.0	(%)	1.7 < S < 1.99	(%)	S < 1.7	(%)		S >2.0	(%)	1.7 < S < 1.99	(%)	S < 1.7	(%)		S >2.0	(%)	1.7 < S < 1.99	(%)	S < 1.7	(%)
**(A)**																												
**Overall**	193	166	86.0	21	10.9	6	3.1	201	179	89.0	18	9.0	4	2.0	177	118	66.7	43	24.3	16	9.0	190	146	76.8	28	14.8	16	8.4
**Gram-Negative (GN)**	84	81	96.4	3	3.6	0	0	91	88	96.7	2	2.2	1	1.1	81	66	81.5	13	16.0	2	2.5	67	63	94.0	2	3.0	2	3.0
**Gram-Positive (GP)**	103	82	79.6	18	17.5	3	2.9	104	87	83.6	14	13.5	3	2.9	88	51	58.0	28	31.8	9	10.2	122	81	67.2	27	21.3	14	11.5
**(B)**																												
**Enterobacteriales**	74	71	95.9	3	4.1	0	0	77	75	97.4	1	1.3	1	1.3	69	58	84.1	10	14.5	1	1.4	51	50	98.0	1	2.0	0	0
Escherichia coli	41	41	100	0	0	0	0	37	36	97.3	1	2.7	0	0	35	28	80.0	6	17.1	1	2.9	36	34	94.4	2	5.6	0	0
***Klebsiella* spp.**	19	18	94.7	1	5.3	0	0	27	27	100	0	0	0	0	15	14	93.3	1	6.7	0	0	9	9	100	0	0	0	0
Pseudomonas	4	4	100	0	0	0	0	5	5	100	0	0	0	0	4	4	100	0	0	0	0	2	2	100	0	0	0	0
**Other GN**	4	4	100	0	0	0	0	9	8	88.9	1	11.1	0	0	8	4	50.0	3	37.5	1	12.5	16	13	81.2	1	6.3	2	12.5
S. aureus	28	28	100	0	0	0	0	20	20	100	0	0	0	0	12	11	91.7	1	8.3	0	0	35	31	88.6	1	2.8	3	8.6
**CoN staphylococci**	44	29	65.9	14	31.8	1	2.3	35	25	71.4	9	25.7	1	2.9	27	16	59.3	11	40.7	0	0	47	21	44.7	19	40.4	7	14.9
Streptococci	15	12	80.0	1	6.7	2	13.3	19	18	94.7	1	5.3	0	0	30	11	36.7	11	36.7	8	26.6	22	16	72.8	3	13.6	3	13.6
Enterococci	5	5	100	0	0	0	0	7	7	100	0	0	0	0	12	11	91.7	1	8.3	0	0	13	13	100	0	0	0	0
**Other GP**	11	9	81.8	2	18.2	0	0	23	17	73.9	4	17.4	2	8.7	7	2	28.6	4	57.1	1	14.3	5	0	0	4	80.0	1	20.0
**Yeast**	6	3	50.0	0	0	3	50.0	6	4	66.7	2	33.3	0	0	8	1	12.5	2	25.0	5	62.5	1	1	100	0	0	0	0

Tabla S1. Performance of the rapid BACpro® II kit in the 4 participant laboratories.

	Laboratory	rapidBACpro® II kit	Score	Sepsityper® kit	Score	Differential Centrifugation Method	Score
Staphylococcus aureus + Enterococcus faecium	OUH	*E. faecium*	2,21	*S.aureus - E.faecium*	1,98 - 1,75	-	-
Capnocytophaga sputigena + Fusobacterium nucleatum	OUH	none	-	*none*	-	-	-
Enterobacter cloacae + Streptococcus cristatus	CHUAC	*E. cloacae*		*S. cristatus*		-	-
Staphylococcus capitis + Staphylococcus hominis	CHUAC	*S. capitis*		*none*		-	-
Klebsiella pneumoniae + Enterococcus faecium + Clostridium perfringens	HGM	*K. pneumoniae*	2,36	*K. pneumoniae*	2,38	*K. pneumoniae*	2,16
Klebsiella pneumoniae + Enterococcus faecium + Aeromonas hydrophila	HGM	*A. hydrophila*	2,08	*A. hydrophila*	2,24	*E. faecium + A. hydrophila*	1,69 - 1,81
Staphylococcus aureus + Staphylococcus epidermidis	HGM	*S. aureus*	2,27	*S. aureus*	2,38	*S. aureus*	1,46
Staphylococcus aureus + Streptococcus mitis	HGM	none	-	*none*	-	none	-
Serratia marcescens + Enterococcus faecium	HGM	*E. faecium*	2,31	*E. faecium*	2,36	*E. faecium*	1,76
Streptococcus parasanguinis + Neisseria sp.	HGM	*S. parasanguinis*	1,85	*S. parasanguinis*	1,97	*S. parasanguinis*	-
Acinetobacter baumannii + Staphylococcus epidermidis	HGM	*A. baumannii*	2,17	*none*	-	*A. baumannii*	2,33
Klebsiella pneumoniae + Serratia marcescens	HGM	*K. pneumoniae*	2,26	*K. pneumoniae*	2,30	*K. pneumoniae*	2,40
Escherichia coli+ Serratia marcescens	HGM	*E. coli*	2,10	*E. coli*	2,26	*E. coli*	2,02
Staphylococcus epidermidis + Staphylococcus capitis	HGM	*S. capitis*	1,93	*S. capitis*	1,81	*S. capitis*	2,23
Staphylococcus epidermidis + Clostridium perfringens	HGM	*C. perfringens*	2,03	*C. perfringens*	2,02	*C. perfringens*	1,96
Klebsiella pneumoniae + Enterococcus faecalis	HGM	*K. pneumoniae*	2,55	*K. pneumoniae*	2,26	*K. pneumoniae*	2,45
Klebsiella pneumoniae + Enterococcus faecalis	HGM	*K. pneumoniae*	2,20	*K. pneumoniae*	2,26	*K. pneumoniae*	2,29
Escherichia coli + Enterococcus faecium	HGM	*E. coli*	1,67	*E. coli*	2,47	*E. coli*	2,13
Enterococcus faecalis + Pseudomonas aeruginosa	HGM	*E. faecalis*	2,23	*E. faecalis*	2,21	*E. faecalis*	2,20
Enterococcus faecium + Staphylococcus epidermidis	HGM	*E. faecium*	2,17	*E. faecium*	2,34	*E. faecium*	2,39
Klebsiella pneumoniae + Bacillus sp.	HGM	*K. pneumoniae*	2,20	*K. pneumoniae*	2,45	*K. pneumoniae*	2,25
Klebsiella pneumoniae + Bacillus sp.	HGM	*K. pneumoniae*	2,17	*K. pneumoniae*	2,28	*K. pneumoniae*	2,08
Staphylococcus epidermidis + Staphylococcus capitis	HGM	*S. epidermidis - S. capitis*	2.08 - 1,77	*S. epidermidis*	1,85	none	-
Escherichia coli + Enterococcus faecalis	HGM	*E. coli*	2,30	*E. coli*	2,25	*E. coli*	2,25
Klebsiella pneumoniae + Staphylococcus haemolyticus	HGM	*K. pneumoniae -S. haemolyticus*	1,87 - 1,85	*S. haemolyticus*	2,14	*S. haemolyticus*	1,71
Enterococcus faecalis + Staphylococcus epidermidis	HGM	*E. faecalis*	1,75	*E. faecalis*	2,27	*E. faecalis*	2,25
Staphylococcus epidermidis + Streptococcus oralis	HGM	*S. oralis*	2,07	*S. oralis*	2,25	none	-

Table S2. Identification of the polymicrobial blood cultures with different methods.
